# Thank you to our reviewers 2022

**DOI:** 10.18849/ve.v8i1.662

**Published:** 2023-02-17

**Authors:** Kit Sturgess+

**Keywords:** THANK YOU

## Abstract

As 2023, dawns we are faced with a very different world than at the start of 2022, COVID-19 is in the rear-view mirror, but we now face the war in Ukraine, energy prices and inflation. Perhaps this is a glimpse of the ‘new world’ where we will be continually challenged by crisis whether it be conflict, infectious disease or the impacts of global warming.

On a more positive note it has been another busy and productive year for *Veterinary Evidence* as we strive to continually improve the journal whether you are a reader, author or reviewer. In April we said ‘goodbye’ to Daniel Edwards who had covered Bridget Sheppard’s maternity leave as Managing Editor. I would like to take the opportunity to thank Dan for all his hard work for *Veterinary Evidence* initiating a number of projects that have come to fruition through the year and to welcome Bridget back. As *Veterinary Evidence* has grown, we have been fortunate to be able to expand our staff to meet the need and ensure efficient turnaround of submissions so another welcome, William Smith has joined the team and is now a permanent full time Editorial Assistant.

It is not just staff changes that happened in 2022, we have also improved the governance of *Veterinary Evidence* with the appointment of new board members and creation of a Deputy Editor-in-Chief position ably filled by Louise Buckley. We also submitted a successful application to COPE (The Committee on Publication Ethics) further strengthening the journal’s oversight and reputation.

We have had a major website redesign, now live, which has greatly improved the look, feel, and functionality of *Veterinary Evidence* for all users. Readers can find relevant papers quickly and efficiently. Our student awards continue to be a success and I would like to thank the whole team of staff and reviewers for turning round these submissions so quickly. I had the pleasure of talking to the prize-winning authors and it was great to hear their enthusiasm but also how much they have valued the help and support from the journal.

We have also been developing our processes for reviewing papers to make sure that papers are ready to go out for review. We are working on ways to make sure that, for papers requiring revision, authors respond to each of the comments made by reviewers, and reviewers can quickly see that there has been an author response to their comments and suggestions. A new submission system, Editorial Manager , has been implemented that should make life easier for everyone as well as provide accessible data on papers as they go through the system so we can see what we do well and where we can do better.

None of this would be possible without the input from our reviewers and I would like to take this opportunity to say a huge thank you to all our reviewers and everyone who has served on the Editorial Board this year for their tireless efforts on behalf of *Veterinary Evidence* – without your support the journal could not exist. This year the Board has again had three online meetings, which have been essential in helping develop the strategy for *Veterinary Evidence* such as how we ensure our Knowledge Summaries remain current, what literature should be included in Knowledge Summaries and how we improve the reviewer and author experience.

Once again, a massive thank you to everyone who works for and supports *Veterinary Evidence*, wishing you all a safe and productive 2023.

Kit Sturgess

Editor-in-Chief

As 2023, dawns we are faced with a very different world than at the start of 2022, COVID-19 is in the rear-view mirror, but we now face the war in Ukraine, energy prices and inflation. Perhaps this is a glimpse of the ‘new world’ where we will be continually challenged by crisis whether it be conflict, infectious disease or the impacts of global warming.

On a more positive note it has been another busy and productive year for *Veterinary Evidence *as we strive to continually improve the journal whether you are a reader, author or reviewer. In April we said ‘goodbye’ to Daniel Edwards who had covered Bridget Sheppard’s maternity leave as Managing Editor. I would like to take the opportunity to thank Dan for all his hard work for* Veterinary Evidence *initiating a number of projects that have come to fruition through the year and to welcome Bridget back. As* Veterinary Evidence *has grown, we have been fortunate to be able to expand our staff to meet the need and ensure efficient turnaround of submissions so another welcome, William Smith has joined the team and is now a permanent full time Editorial Assistant.

It is not just staff changes that happened in 2022, we have also improved the governance of* Veterinary Evidence* with the appointment of new board members and creation of a Deputy Editor-in-Chief position ably filled by Louise Buckley. We also submitted a successful application to COPE *(*The Committee on Publication Ethics) further strengthening the journal’s oversight and reputation.

We have had a major website redesign, now live, which has greatly improved the look, feel, and functionality of *Veterinary Evidence *for all users. Readers can find relevant papers quickly and efficiently. Our student awards continue to be a success and I would like to thank the whole team of staff and reviewers for turning round these submissions so quickly. I had the pleasure of talking to the prize-winning authors and it was great to hear their enthusiasm but also how much they have valued the help and support from the journal.

We have also been developing our processes for reviewing papers to make sure that papers are ready to go out for review. We are working on ways to make sure that, for papers requiring revision, authors respond to each of the comments made by reviewers, and reviewers can quickly see that there has been an author response to their comments and suggestions. A new submission system, Editorial Manager , has been implemented that should make life easier for everyone as well as provide accessible data on papers as they go through the system so we can see what we do well and where we can do better.

None of this would be possible without the input from our reviewers and I would like to take this opportunity to say a huge thank you to all our reviewers and everyone who has served on the Editorial Board this year for their tireless efforts on behalf of *Veterinary Evidence* – without your support the journal could not exist. This year the Board has again had three online meetings, which have been essential in helping develop the strategy for *Veterinary Evidence* such as how we ensure our Knowledge Summaries remain current, what literature should be included in Knowledge Summaries and how we improve the reviewer and author experience.

Once again, a massive thank you to everyone who works for and supports *Veterinary Evidence*, wishing you all a safe and productive 2023.

Kit Sturgess

Editor-in-Chief

Fergus Allerton

Isabel Amores-Fuster

Davina Anderson

Jonathan Anderson

Judy Bettridge

Paul Bloom

Jenny Brown

Louise Buckley

Fernanda Camacho

John Campbell

Deirdre Campion

Tom Candy

John Carr

Marios Charalambous

Tim Charlesworth

John Chitty

Louise Clark

Jacqueline Cole

Francesca Compostella

Michelle Dawson

Nicole deToit

Jane Dobson

Jacklyn Ellis

Nathan Erickson

Erik Fausak

John Fishwick

Aaron Fletcher

Barny Fraser

Mary Fraser

Merran Govendir

Mathilde Granger

Kathryn Griffiths

Danielle Gunn-Moore

Matthew Gurney

Emily Hall

Alastair Hayton

Guy Hinnigan

Jodie Hughes

Elizabeth Jackson

Simone Kirby

Clare Knottenbelt

David Leicester

William McFadzean

Anna Meredith

Tristan Merlin

Joanna Morris

Andy Morris

Sue Murphy

Christopher Norkus

Conor O'Halloran

Maureen O'Mara

Catrina Pennington

Roberta Perego

Kiro Petrovski

Robert Pettitt

Simon Platt

David Ramey

Ian Ramsey

Nicki Reed

Kerry Rolph

Silke Salavati

Richard Saunders

Rebecca Schofield

Ellen Singer

Fiona Smith

Lesca Sofyan

Eva Spada

Fabio Stabile

Jonathan Statham

Essa Suleman

Adam Swallow

Severine Tasker

Theodora Tsouloufi

Hélène Vandenberghe

Nick Wojciechowski

Najat Yahia

Maurice Zandvliet

## Disclaimer

Veterinary Evidence is a resource to help inform, and the content herein should not override the responsibility of the practitioner.

Practitioners should also consider factors such as individual clinical expertise and judgement along with patient’s circumstances and owners’ values.

Authors are responsible for the accuracy of the content.

While the Editor and Publisher believe that all content herein is in accord with current recommendations and practice at the time of publication, they accept no legal responsibility for any errors or omissions, and make no warranty, express or implied.

For further information please refer to our Terms of Use.

